# Potassium deficiency causes more nitrate nitrogen to be stored in leaves for low-K sensitive sweet potato genotypes

**DOI:** 10.3389/fpls.2022.1069181

**Published:** 2022-11-23

**Authors:** Jingran Liu, Houqiang Xia, Yang Gao, Dongyu Pan, Jian Sun, Ming Liu, Zhonghou Tang, Zongyun Li

**Affiliations:** ^1^ Institute of Integrative Plant Biology, School of Life Sciences, Jiangsu Normal University, Xuzhou, China; ^2^ Jiangsu Key Laboratory of Phylogenomics and Comparative Genomics, School of Life Sciences, Jiangsu Normal University, Xuzhou, China; ^3^ Xuzhou Institute of Agricultural Sciences of Xuhuai District of Jiangsu Province, Xuzhou, China

**Keywords:** sweet potato (*Ipomoea batatas*), potassium deficiency, N metabolism, nitrate, leaf K

## Abstract

In order to explore the effect of potassium (K) deficiency on nitrogen (N) metabolism in sweet potato (*Ipomoea batatas* L.), a hydroponic experiment was conducted with two genotypes (Xushu 32, low-K-tolerant; Ningzishu 1, low-K-sensitive) under two K treatments (−K, <0.03 mM of K^+^; +K, 5 mM of K^+^) in the greenhouse of Jiangsu Normal University. The results showed that K deficiency decreased root, stem, and leaf biomass by 13%–58% and reduced whole plant biomass by 24%–35%. Compared to +K, the amount of K and K accumulation in sweet potato leaves and roots was significantly decreased by increasing root K^+^ efflux in K-deficiency-treated plants. In addition, leaf K, N, ammonium nitrogen (NH_4_^+^–N), or nitrate nitrogen (NO_3_^−^–N) in leaves and roots significantly reduced under K deficiency, and leaf K content had a significant quadratic relationship with soluble protein, NO_3_^−^–N, or NH_4_^+^–N in leaves and roots. Under K deficiency, higher glutamate synthase (GOGAT) activity did not increase amino acid synthesis in roots; however, the range of variation in leaves was larger than that in roots with increased amino acid in roots, indicating that the transformation of amino acids into proteins in roots and the amino acid export from roots to leaves were not inhibited. K deficiency decreased the activity of nitrate reductase (NR) and nitrite reductase (NiR), even if the transcription level of *NR* and *NiR* increased, decreased, or remained unchanged. The NO_3_^−^/NH_4_^+^ ratio in leaves and roots under K deficiency decreased, except in Ningzishu 1 leaves. These results indicated that for Ningzishu 1, more NO_3_^−^ was stored under K deficiency in leaves, and the NR and NiR determined the response to K deficiency in leaves. Therefore, the resistance of NR and NiR activities to K deficiency may be a dominant factor that ameliorates the growth between Xushu 32 and Ningzishu 1 with different low-K sensitivities.

## Introduction

IntroductionPotassium (K) is one of the necessary nutrients and the most crucial osmotic factor in crops (Wang et al., 2016a). Previous studies have shown that K can improve crop stress resistance by optimizing gas exchange, stomatal conductance, protein synthesis, enzyme activation, and photochemical transport (Wang et al., 2013; Zahoor et al., 2017a; Zahoor et al., 2017b). Statistically, at present, about 60% of the total cultivated land area was short of K in China, among which the serious shortage of K (available K < 70 mg kg^−1^) occupies 22.6% of the total cultivated land area (Xia, 2016). In China, about 70% or more of domestic potash relies on imports for a long time. The high price of potash led to farmers’ reluctance to use potash, and the shortage of K in cultivated land becomes increasingly serious (Wang et al., 2012). With the increasing shortage of potash resources and the increasingly serious situation of soil K deficiency, the yield and quality decline of sweet potato tuber, caused by soil K deficiency, will be increasingly aggravated, which has become one of the important reasons to restrict the tuber production in sweet potato.

Nitrogen (N), as the basic substance of nucleotide, amino acid (AA), and protein, is also a crucial factor in plant growth and development. Nitrate nitrogen (NO_3_^−^–N) and ammonia nitrogen (NH_4_^+^–N) are the main forms of N absorption, rather than AAs or other organic forms of N. It has been found that adequate K is an indispensable condition for normal N metabolism. K fertilizer significantly affected the accumulation of K and N in sweet potato tuber (George et al., 2002), and the distribution rate of shoot ^15^N was significantly increased during the tuber formation period (Wang et al., 2016b). In addition, K can also significantly enhance the activity of nitrate reductase (NR), which is conducive to the absorption, transport, and reduction of NO_3_^−^–N in crops and promote protein synthesis rate (Hu et al., 2016). It was observed that K^+^ has a close relationship with NO_3_^−^–N uptake by roots and affects photosynthetic production, thus affecting the NO_3_^−^–N active absorption process (Wang et al., 2016b). Moreover, in the xylem, K is transported with NO_3_^−^–N together and affects NO_3_^−^–N distribution between roots and leaves (Ruiz and Romero, 2002; Hu et al., 2016). NH_4_^+^–N could affect root NO_3_^−^–N transport and thus play a main role in the regulation of N absorption by the crops (Nacry et al., 2013). Under abiotic stress, NH_4_^+^ produced through NR and nitrite reductase-mediated (NiR) reduction of NO_3_^−^ was reduced as well as the activities of glutamine synthetase (GS) and glutamate synthase (GOGAT). These results led to NH_4_^+^ accumulation and toxic effects on plants (Cao et al., 2009). However, in sweet potatoes, it is not clear that the changes in related enzymes and genes are due to K deficiency.

K deficiency significantly increased AA and soluble protein in cotton leaves (Wang et al., 2012). In contrast, AA and protein were reduced in leaves of cucumber (*Cucumis sativus*) and corn (*Zea mays* L.) (Ruiz and Romero, 2002; Qu et al., 2011) or had an opposite trend in the subtending leaf to cotton boll (Hu et al., 2016). Under K deficiency, AA and protein differ between crop species and varieties. Sweet potato is widely cultivated in China for its high stability and wide adaptability and has higher K requirements for optimum yield than cereals and oilseeds, followed by N and phosphorus (Tang et al., 2015). Previous studies have reported that K application increased chlorophyll content and net photosynthetic rate in sweet potato leaves (Chen et al., 2013), stimulated sucrose-to-starch conversion and N accumulation, and finally promoted starch accumulation and storage root yield (Wang et al., 2016b; Wang et al., 2017; Gao et al., 2021). Under hydroponic conditions, K deficiency suppressed biomass accumulation in blades, petioles, and roots in all three cultivars with low K-use efficiency, high K-uptake efficiency, and high K-use efficiency, and impaired phloem loading due to K deficiency associated with a decline in photosynthetic rate and decreased carbohydrate supply from blades, resulting in restricted root growth (Wang et al., 2018). However, how K deficiency regulates N metabolism in the leaves and roots of two sweet potato cultivars with a significant difference in K sensitivity has not been reported yet. Therefore, in this study, we want to 1) detect the K content and K^+^ efflux, N, NO_3_^−^–N and NH_4_^+^–N, soluble protein, and AA in leaves and roots under K deficiency with the purpose of determining the relationship of leaf K with N metabolism in Xushu 32 and Ningzishu 1; 2) measure the enzyme activities and gene transcription levels in order to filter N-metabolizing key enzymes to low K in Xushu 32 and Ningzishu 1 with different levels of low K sensitivity; and 3) clarify the differences between Xushu 32 and Ningzishu 1 in N metabolism for leaves and roots in response to K deficiency.

## Materials and methods

### Plant materials and K treatment

Sweet potato cultivars were different and sensitive to potassium (K). Based on the variance of the K sensitivity index, 31 good-quality sweet potato cultivars (lines) were studied under the NP plot (treatment of nitrogen and phosphorus) and NPK plot (treatment of nitrogen, phosphorus, and K) (Tang et al., 2014). Sweet potato cultivars (lines) were clustered into four groups: K-high-sensitive group (typical for Ningzishu 1), K-sensitive group (typical for Beijing 553), K-moderate-sensitive group (typical for Xushu 22), and K-tolerance group (typical for Xushu 32). Therefore, Xushu 32 (low-K-tolerant) and Ningzishu 1 (low-K-sensitive) were used in this study. Furthermore, Gao et al. (2021) found that Ningzishu 1 was more sensitive than Xushu 32 in tuber sucrose-to-starch conversion under different K applications, too (Gao et al., 2021).

The shoots of Xushu 32 were obtained from the laboratory of Tang Zhonghou, Xuzhou Academy of Agricultural Sciences. The shoots of Ningzishu 1 were obtained from the Laboratory of Xie Yizhi at Jiangsu Academy of Agricultural Sciences. Shoots with five leaves were collected from tuberous root (20 days), then arranged into transfer pots (12 plants/plot), and cultivated with 8 L (40 × 23 × 12 cm) of 1/4-strength Hoagland solution (Xia et al., 2020). The solution was renewed every 48 h. After a pretreatment period of 5 days, seedlings were divided into two groups with the following treatments: K deficiency (−K, <0.03 mM of K^+^) and K sufficiency (+K, 5 mM of K^+^). The amount of K^+^ was varied by changing the amount of K_2_SO_4_. Seedlings were grown under a condition with a photoperiod of 16 h, a photosynthetic flux density of 150 mmol m^−2^ s^−1^, and temperatures at 20°C–25°C. The condition was repeated in every pot, and six pots were grown for each treatment.

At 0, 5, 10, and 15 days of K treatment (DKT), functional leaves (youngest fully expanded main-stem leaf) and fine roots were collected for analysis of N-metabolizing enzymes, AA and protein, NO_3_^−^–N and NH_4_^+^–N, N and K contents, and steady-state root K^+^ fluxes.

### K content, N content and K^+^ flux assay

Oven-dried samples (roots, stems, and leaves) of 0.1 g through a 0.25-mm sieve were digested using 5 mL of H_2_SO_4_–H_2_O_2_ to analyze K and N contents. The K content was measured by flame photometry, while N was measured with the automated discrete analyzer (SmartChem 200, AMS Alliance, Rome, Italy) (Gao et al., 2019). The steady-state net K^+^ fluxes in sweet potato roots at 10 DKT were measured non-invasively by the NMT system (NMT-100-SIM-YG, Younger USA LLC, Amherst, MA, USA) according to previously described methods (Sun et al., 2009; Yu et al., 2016; Liu et al., 2019). The K^+^ concentration of K^+^-specific microelectrode followed standard procedures. Fluxes were automatically recorded in the apex region and the mature region.

### NO_3_^−^–N, NH_4_^+^–N, and amino acid content

Oven-dried samples (roots and leaves) of 0.2 g were mixed with distilled water (10 mL) at 100°C for 1 h to analyze NO_3_^−^–N and NH_4_^+^–N. NO_3_^−^–N was measured using a salicylic acid method (Ruiz and Romero, 2002). NH_4_^+^–N was measured using a colorimetric method (Weatherburn, 1967; Xia et al., 2020). In addition, AA content was measured using the acid ninhydrin (Liu et al., 2015).

### Enzyme extraction and analysis

NR and NiR in leaves and roots were determined according to previous studies (Yu et al., 2016; Xia et al., 2020). Fresh tissue of leaves and roots (0.3 g) was ground with 4 mL of 0.1 M phosphate buffer (pH 7.5). The activities of NR and NiR were measured by the light absorption of residual NO_2_^−^ at 540 nm. The protein content was analyzed by G-250 reagent using bovine serum albumin as a standard (Bradford, 1976).

GS and GOGAT were extracted and analyzed in leaves and roots, according to previous studies (Barbosa et al., 2010; Xia et al., 2020). GS activity was analyzed using the formation quantity of γ-glutamylhydroxamate. GOGAT activity was measured using a microplate reader at 340 nm by producing NADH oxidation.

### Transcriptional level analysis of genes

The total RNA from control and treated roots and leaves at 15 DKT was extracted with the use of a DP441-50T RNAprep pure plant kit from Tiangen Biotech (Beijing, China) according to the manufacturer’s instructions. The total RNA (2 μg) was reverse-transcribed using a PrimeScript RT reagent cDNA kit (Takara). Afterward, the synthetic cDNA was used as a template for real-time PCR amplification. The primers of related genes were synthesized by Sangon Biotechnology (Shanghai, China), and the sequences were shown in **Supplementary Table 1**. *GAPDH* was used as a reference gene, and the relative transcriptional levels of NRT1.1, NR, NiR, GS, and GOGAT were calculated using the 2^−ΔΔCT^ method (Park et al., 2012).

### Statistical analysis

Data were analyzed with Origin 2018 and SPSS 23.0, and the results in the figures and tables are shown as the average value ± SE (n ≥ 3). Statistical analysis used Tukey’s honestly significant difference (HSD) test (*p* < 0.05).

## Results

### Effects of K deficiency on biomass and K and N accumulation in sweet potato seedlings

**Figure 1:**
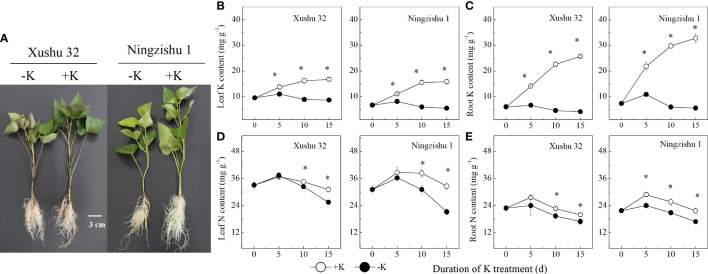
Difference in the sensitivity to K deficiency between the two sweet potato genotypes (low-K-tolerant “Xushu 32” and low-K-sensitive “Ningzishu 1”). **(A)** Phenotypic difference of sweet potato plants during K deficiency (−K) and K sufficient (+K) treatments for 10 days. **(B, C)** leaf and root K contents. **(D, E)** Leaf and root N contents. The stages labeled with an asterisk (*) indicate significant differences (*p* < 0.05) between −K and +K.

Compared with the +K treatment (+K), plant growth inhibition was observed in the −K treatment (−K, **Figure 1A**), leading to a significant decline in biomass under K deficiency (**Table 1**). For Xushu 32 and Ningzishu 1, K deficiency reduced plant biomass by 24% and 35%, respectively. Ningzishu 1 was more drastically affected in the biomass of root and leaf when compared with Xushu 32, the biomass of root and leaf declined by 58% and 33% in −K than that in +K for Ningzishu 1, respectively, and a smaller decrease of 14%–20% was observed for Xushu 32. In addition, K deficiency significantly decreased the root–shoot ratio of Ningzishu 1 by 41% but had no significant effect on that of Xushu 32. Additionally, the K and N accumulation per plant also varied significantly between +K and −K and decreased by 79%–87% and 34%–56% in −K, respectively. The amplitude of variations of Ningzishu 1 for K and N accumulation were greater than those of Xushu 32.

**Table 1:** Growth parameters, K accumulation, and N accumulation of sweet potato seedlings as affected by the different K treatments.

Cultivar	K treatment	Biomass (g per plant)				Root–shoot ratio	K and N accumulation (g per plant)	
		Root	Stem	Leaf	Plant		K	N
Xushu 32	+K	0.72a	1.63a	1.43a	3.79a	0.24b	81.05a	73.70a
	−K	0.62a	1.10b	1.15ab	2.87b	0.28ab	17.32c	48.64b
Ningzishu 1	+K	0.63a	0.62c	0.98bc	2.23bc	0.38a	53.89b	54.40b
	−K	0.27b	0.54c	0.66c	1.46c	0.22b	7.21c	23.96c
Significance analysis								
Cultivar (C)		ns	**	**	**	ns	**	**
K treatment (K)		*	**	*	*	ns	**	**
C×K		ns	*	ns	ns	*	*	ns

Values followed by a different letter within the same column including Xushu 32 and Ningzishu 1 are significantly different at 0.05 level.

* and ** mean significant at the p < 0.05 and 0.01 probability levels, respectively; ns means non-significant.

### Effects of K deficiency on K and N content in sweet potato leaves and roots

#### K and N contents

In −K, the content of K in leaves and roots increased firstly, then decreased with DKT, and reached the peak value at 5 DKT (**Figures 1B, C**). Compared to +K, the content of leaf K and root K was lower in −K for Xushu 32 and Ningzishu 1 at each sampling point, and the difference increased with DKT. At 15 DKT, the average content of leaf K and root K in −K reduced by 20%–27% and 84%–85% for both cultivars, respectively.

The N content in leaves and roots increased firstly, then decreased with DKT, and reached the peak value at 5 DKT (**Figures 1D, E**). Compared to +K, the N content in leaves and roots for Ningzishu 1 and Xushu 32 decreased significantly in −K. When all data for each K treatment were averaged, the leaf N content and root N content in −K declined by 5%–11% for Xushu 32 and approximately 15% for Ningzishu 1, respectively.

**Figure 2:**
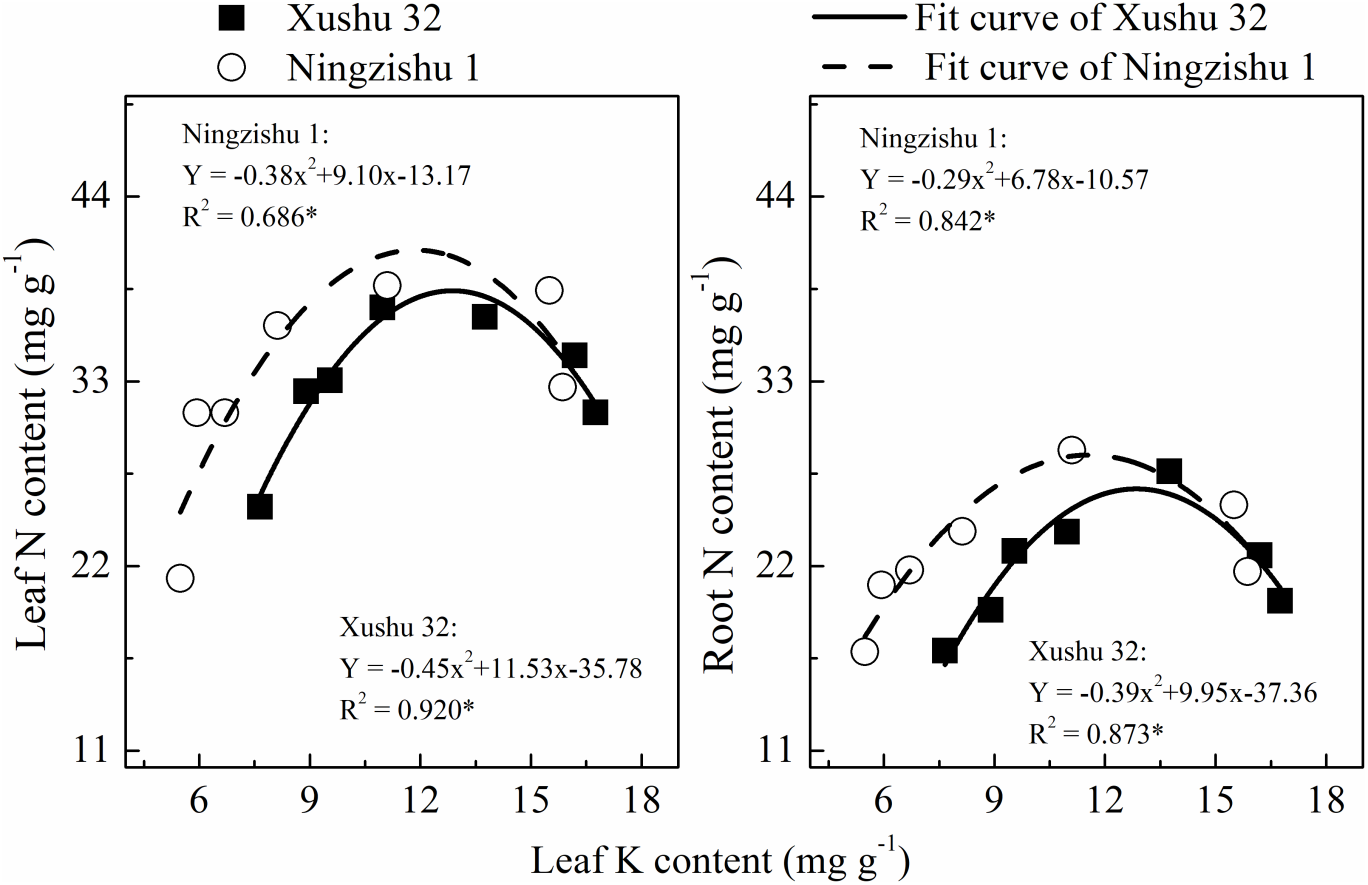
Relationship between leaf nitrogen (N) content, root N content, and leaf potassium (K) content. The solid and dotted lines represent Xushu 32 and Ningzishu 1, respectively; * mean significant at the *p* < 0.05 probability level.

The N content in leaves and roots was fitted with a quadratic equation (R^2^ = 0.686–0.920*, **Figure 2**). With the increase of leaf K content, the N content in leaves and roots showed a downward opening parabolic trend. For Ningzishu 1, the degree of opening and the maximum value of the fitting equation was larger than that for Xushu 32, indicating that the N content in leaves and roots for Ningzishu 1 was more likely to be affected by leaf K content.

#### Root K^+^ steady-state ion fluxes

**Figure 3:**
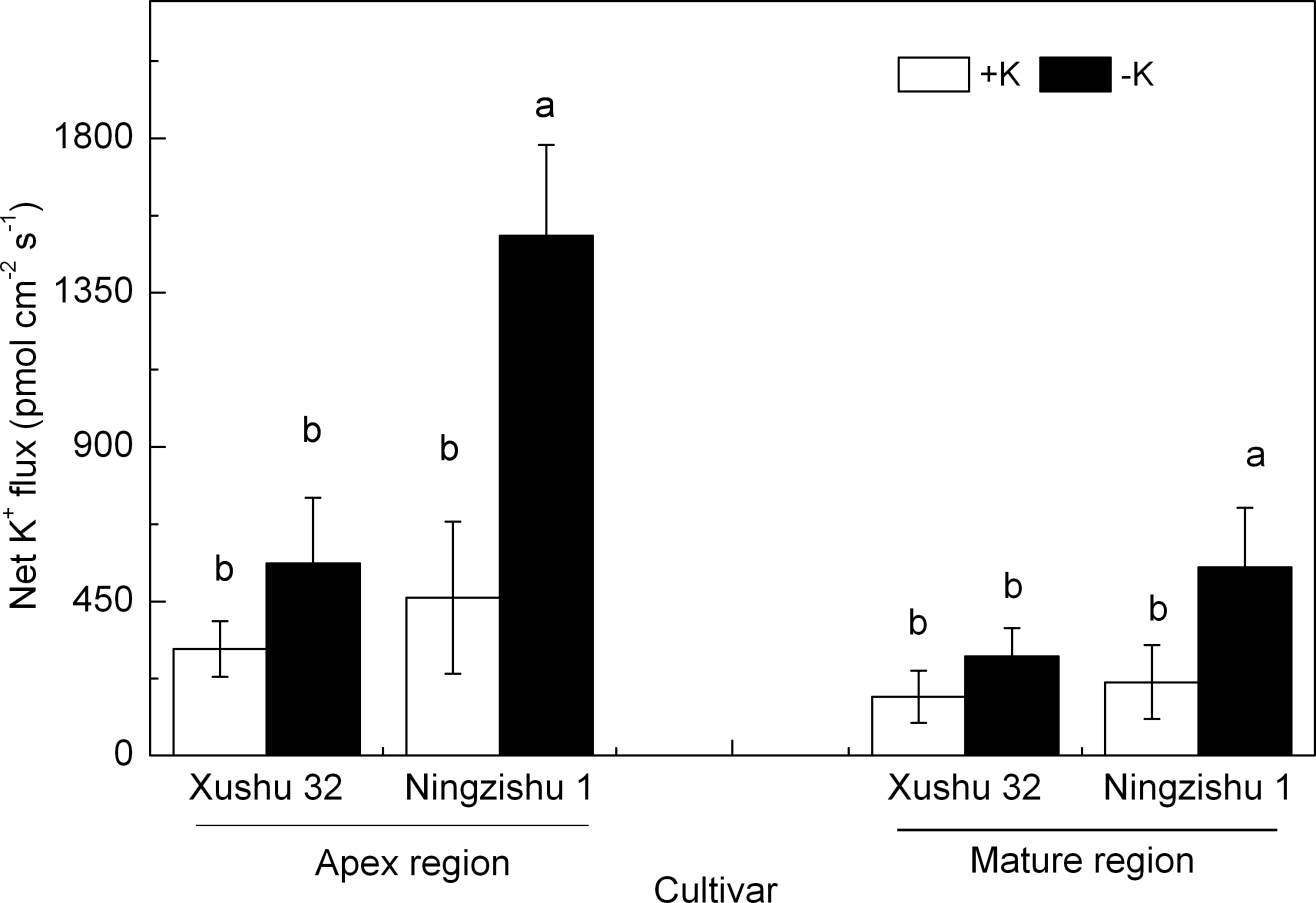
Effects of K deficiency on steady-state net K^+^ efflux in adventitious roots of two sweet potato genotypes (Xushu 32 and Ningzishu 1). Steady-state net K^+^ flux measured from root apex and mature regions after 10 days of K treatment. Apex region, 500 μm from the root tip; mature region, 15 mm from the root tip. Columns labeled with different letters in the same region indicate significant differences at 0.05 level.

At 10 DKT, the K^+^ efflux in the measured regions of Ningzishu 1 roots was significantly stimulated in −K, while the amplitude of K^+^ efflux in Xushu 32 roots was not significantly changed (**Figure 3**). Compared with +K, for Xushu 32 roots, the K^+^ efflux in the apex and mature regions in −K was improved by 80% and 69%, respectively, and those for Ningzishu 1 roots were significantly improved by 2.3-fold and 1.6-fold, respectively.

### Effects of K deficiency on amino acid, soluble protein, NO_3_^−^–N, and NH_4_^+^–N in sweet potato leaves and roots

**Figure 4:**
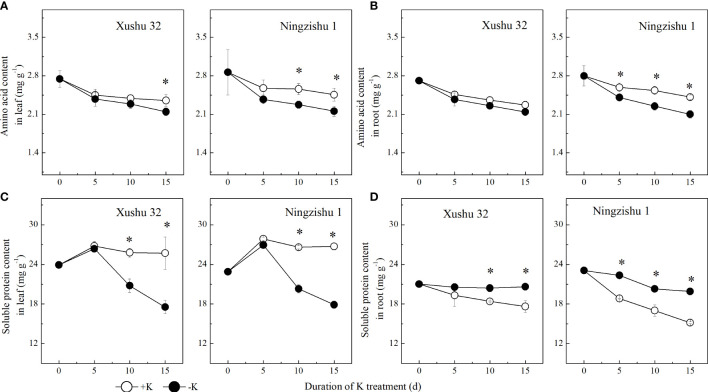
Changes of amino acid and soluble protein content in leaves and roots for two K treatments for Xushu 32 and Ningzishu 1. **(A, B)** leaf and root amino acid contents. **(C, D)** Leaf and root soluble protein contents. The stages labeled with an asterisk (*) indicate significant differences (*p* < 0.05) between −K and +K.

The AA content in leaves and roots declined with DKT (**Figures 4A, B**) in the +K and −K treatments. The difference between −K and +K for AA content increased with DKT, but the significant difference in Ningzishu 1 was bigger than that in Xushu 32. With DKT, the soluble protein content in sweet potato leaves showed a single peak curve, and the peak value appeared at 5 DKT, while the root soluble protein content showed a decreasing trend (**Figures 4C, D**). K deficiency decreased soluble protein content in sweet potato leaves. Compared with +K, in −K, the soluble protein content in Xushu 32 and Ningzishu 1 leaves increased by 32% and 33% at 15 DKT, respectively. Nevertheless, in −K, the soluble protein content in roots showed an opposite trend with an increase of 17%–31% at 15 DKT, which was less than that of the soluble protein in leaves.

**Figure 5:**
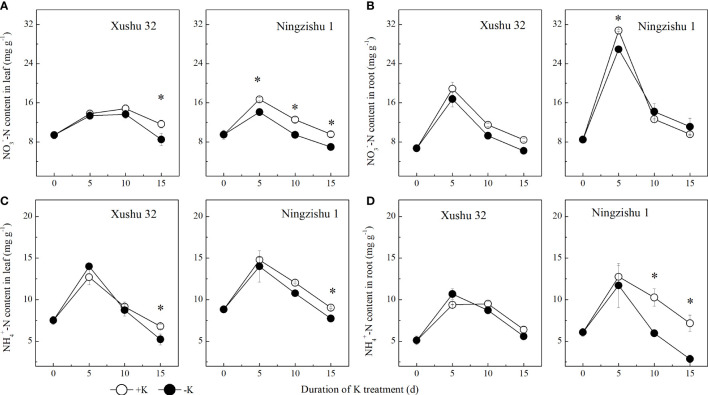
Changes of NO_3_^−^–N and NH_4_^+^–N content in leaves and roots for two K treatments for Xushu 32 and Ningzishu 1. **(A, B)** leaf and root NO_3_^−^–N contents. **(C, D)** Leaf and root NH_4_^+^–N contents. The stages labeled with an asterisk (*) indicate significant differences (*p* < 0.05) between −K and +K.

NO_3_^−^–N content and NH_4_^+^–N content in leaves and roots showed a single-peak curve with DKT, and the peak value appeared at 5 DKT (**Figure 5**). In Xushu 32, the NO_3_^−^–N content and NH_4_^+^–N content in leaves only showed significant levels at 15 DKT (*p* < 0.05), while the two indexes in roots were not significantly affected by K deficiency (*p* > 0.05). In Ningzishu 1, the NO_3_^−^–N content in leaves and NH_4_^+^–N content in roots decreased significantly under K deficiency (*p* < 0.05), and the difference between −K and +K increased with DKT; in particular, the NH_4_^+^–N content in roots was decreased by 8%–60%.

**Figure 6:**
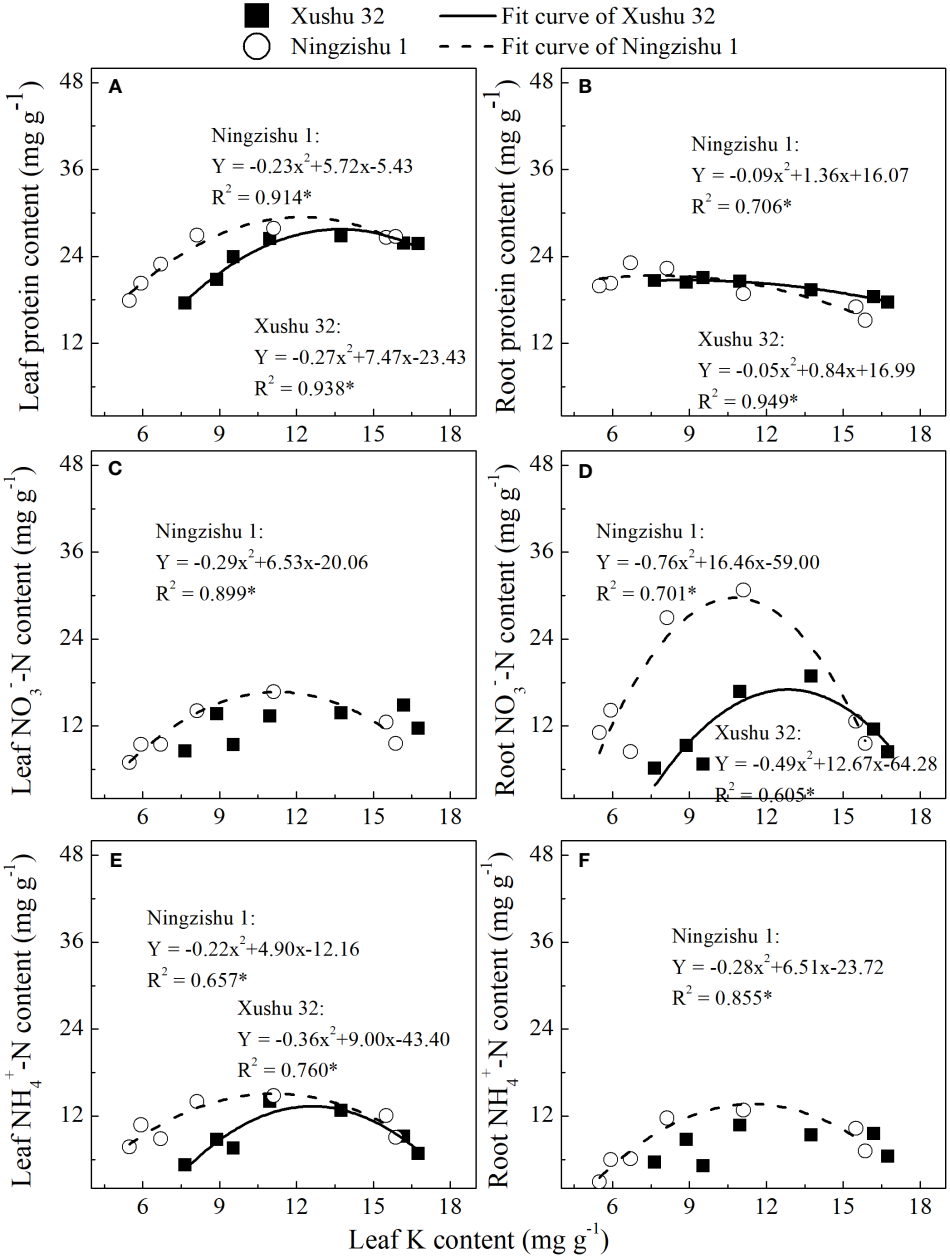
Relationship of protein **(A**, **B)**, NO_3_^−^–N content **(C**, **D)**, and NH_4_^+^–N content **(E**, **F)** in leaves and roots with leaf K content. * mean significant at the p < 0.05 probability level. The solid and dotted lines represent Xushu 32 and Ningzishu 1, respectively.

Except for NO_3_^−^–N in leaves and NH_4_^+^–N in roots for Xushu 32, the content of soluble protein, NO_3_^−^–N, and NH_4_^+^–N in leaves and roots was fitted with quadratic equation (R^2^ = 0.657–0.914*, **Figures 6A–F**). With the increase of leaf K content, the protein, NO_3_^−^–N, and NH_4_^+^–N in leaves and roots showed downward opening parabolic trends.

### Effects of K deficiency on N-metabolizing enzymes in sweet potato leaves and roots

**Figure 7:**
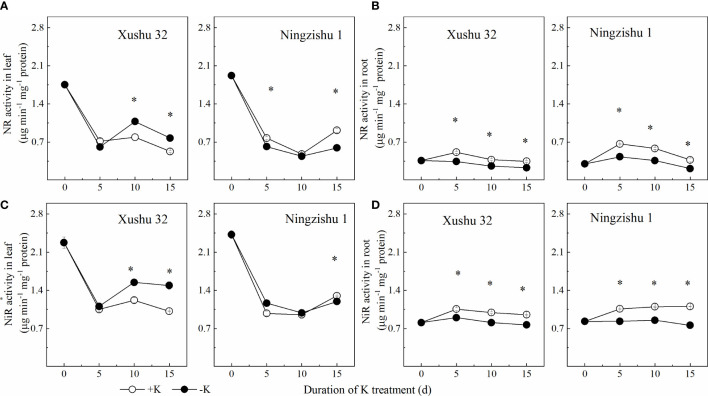
Changes of NR and NiR activities in leaves and roots for two K treatments for Xushu 32 and Ningzishu 1. **(A, B)** leaf and root NR activities. **(C, D)** Leaf and root NiR activities. The stages labeled with an asterisk (*) indicate significant differences (*p* < 0.05) between −K and +K. NR, nitrate reductase; NiR, nitrite reductase.

K deficiency reduced the activities of NR and NiR in leaves and roots for Ningzishu 1 (**Figure 7**). In Ningzishu 1, compared with +K, K deficiency significantly decreased NR activity and NiR activity by 35% and 8% in leaves at 15 DKT (*p* < 0.05), respectively, and by 45% and 30% in roots, respectively. However, for Xushu 32, only the root NR activity was significantly decreased (*p* < 0.05, **Figure 7B**).

**Figure 8:**
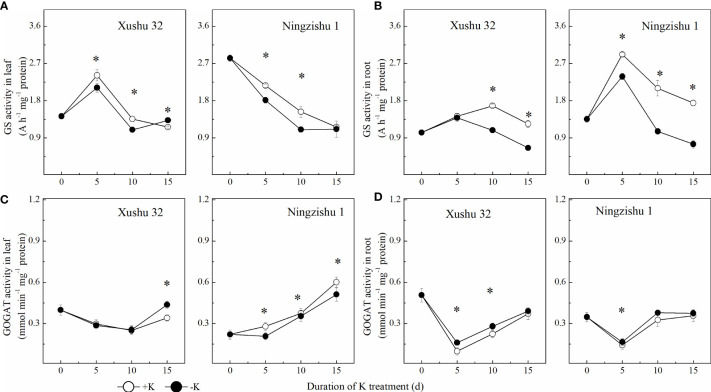
Changes of GS and GOGAT activities in leaves and roots for two K treatments for Xushu 32 and Ningzishu 1. **(A, B)** leaf and root GS activities. **(C, D)** Leaf and root GOGAT activities. The stages labeled with an asterisk (*) indicate significant differences (*p* < 0.05) between −K and +K. GS, glutamine synthetase; GOGAT, glutamate synthase.

GS activity showed a trend of increasing initially and then decreasing with DKT (**Figure 8A**). In Ningzishu 1, compared with +K, leaf GS activity in K-deficiency-treated plants decreased significantly from 5 DKT, with a decline of 13%–20%, but root GS activity decreased significantly from 10 DKT, with a decline of 35%–48% (*p* < 0.05). The GS activity in leaves and roots for Xushu 32 in −K had values similar to those for Ningzishu 1, but the decrements were smaller than those for Ningzishu 1.

With DKT, the changes in GOGAT activity were different from those of GS, presenting a “V” type. Compared with +K, the leaf GOGAT activity treated with −K reduced significantly, while the variation trend of root GOGAT activity was the opposite (**Figure 8C**). At the same DKT, the variation range of Ningzishu 1 under K deficiency was larger than that of Xushu 32.

### Transcriptional levels of genes related to N metabolism under K deficiency

**Figure 9:**
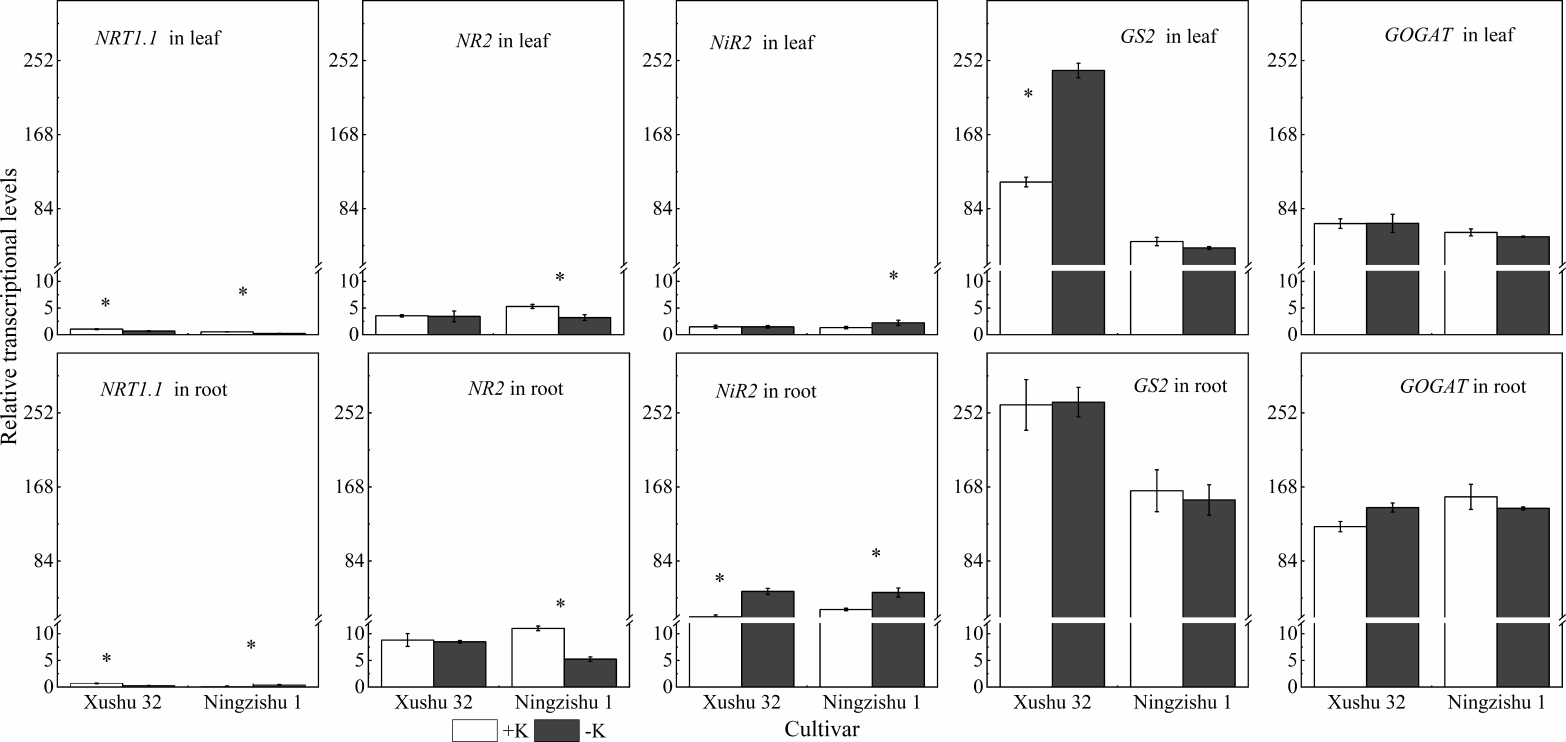
Effects of K deficiency on the transcription of N metabolism-related genes in leaves and roots of two sweet potato cultivars. Sweet potato seedlings were subjected to 15 days of K deficiency stress; total RNA was isolated from leaves and roots for real-time PCR analysis. The stages labeled with an asterisk (*) indicate significant differences (*p* < 0.05) between −K and +K.

*NRT1* involved in nitrate transport in leaves and roots was downregulated in −K, except in Ningzishu 1 roots (**Figure 9**). Under K deficiency, for Ningzishu 1, the transcript levels of *NR2* in leaves and roots were downregulated by 39% and 53%, respectively. However, gene *NR2* in Xushu 32 leaves and roots was not affected by K deficiency. The transcript levels of *NiR* significantly increased in leaves and roots, except in Xushu 32 roots. The −K induced a 69% upregulation in the transcript abundance of *NiR* in leaves and a 67% to 1.4-fold increase in the *NiR* gene transcript abundance in roots. In addition, there were no significant differences in the transcript levels of *GS2* and *GOGAT* in leaves and roots, except in Xushu 32 leaves.

## Discussion

### Effects of K deficiency on biomass and K and N accumulation of sweet potato seedlings

Potassium, as one of the main nutrients, participates in some physiological and biochemical processes in the plant (Wang et al., 2017; Zahoor et al., 2017a). The N content in leaves and roots showed a quadratic response to K content (**Table 1**; **Figure 2**), suggesting that K deficiency affected N metabolism in sweet potatoes, as previously reported in cotton (*Gossypium hirsutum* L.) (Hu et al., 2016). Gao et al. (2021) reported that K affected starch-sucrose metabolism in tuberous roots; in this study, K deficiency increased K^+^ efflux from sweet potato roots, resulting in the decline of K content and accumulation in roots, stems, leaves, and the whole plants (**Table 1**; **Figures 1**, **3**). These results indicated that K deficiency repressed K absorption in the roots of sweet potatoes and abated long-distance K transport from roots to stems to leaves, and similar results were reported in cotton (Hu et al., 2016). Previous studies reported that K deficiency could decrease the yield of sweet potato tuber and potato tuber (Wang et al., 2017; Koch et al., 2019; Gao et al., 2021). In this study, compared with +K, similar results were observed in root biomass and whole plant biomass, resulting in a lower root–shoot ratio (**Table 1**).

### Effects of K deficiency on N assimilation and recycling

In general, abiotic stress reduced plant biomass, coupled with the downregulated expression of genes, which mediated NO_3_^−^ transport, such as *NRT1* (Huang et al., 2018). In this study, K deficiency significantly inhibited the expression of *NRT1* in sweet potatoes, except in Ningzishu 1 roots (**Figure 9**). It was proved that, under abiotic stress, the transcription of *NRT1.1* was mainly repressed in a N-efficient cultivar but was induced in the N-inefficient cultivar (Duan et al., 2016). As is well known, Xushu 32 is tolerant to low K, and Ningzishu 1 is sensitive to low K (Tang et al., 2014; Gao et al., 2021). Therefore, we hypothesized that the difference in *NRT1* gene in K-deficient plants had a consistent performance on Xushu 32 and Ningzishu 1, like N-efficient and N-inefficient cultivars. In future work, this speculation needs to be further verified.

Compared with +K, the NO_3_^−^–N content and NH_4_^+^–N content in sweet potato leaves and roots were significantly reduced under K deficiency (**Figure 5**), suggesting that K could significantly affect N metabolism in sweet potato leaves and roots, and leaf K content is necessary to maintain former activities at an optimum level, consistent with previous results (Hu et al., 2016; Ali et al., 2019). In this study, as exhibited in the relationship in NO_3_^−^–N with leaf K content (**Figure 6**), leaf K at an optimum level had a positive correlation with NO_3_^−^–N, probably because K^+^ had a cooperative transport relationship with NO_3_^−^–N in the xylem from roots to other parts in plants (Xu et al., 2012; Ma et al., 2020), but the excessive leaf K caused the decline of NO_3_^−^–N and NH_4_^+^–N in both leaves and roots. Nevertheless, some research found that low K decreased leaf NO_3_^−^–N content in cotton, and leaf NO_3_^−^–N had a linear positive relationship with leaf K (Hu et al., 2016), inconsistent with this study. Mainly because the crops are different, their samples were gathered from different parts, and their cotton plants were sown in the soil; however, the sweet potato seedlings in this study were planted under hydroponic conditions.

Nitrogen-containing compounds in plants, such as proteins and AAs, are the dominant products of NO_3_^−^ assimilation. In this study, reduced NO_3_^−^–N content and NH_4_^+^–N content indicated that N metabolism could be reduced under K deficiency in leaves and roots, with a significant decrease in AA content (**Figures 4A, B**). In Ningzishu 1, K deficiency significantly decreased AA content in leaves and roots by 7%–13%, and the reduction of AAs in roots was similar to that in leaves; in Xushu 32, the reduction of AAs in leaves at 15 DKT was 9% greater than that in roots (6%). It may be that K deficiency decreased the unloading rate of AAs in the phloem for low-K-tolerant cultivars (Wang et al., 2012), which would decrease AA output from roots to leaves (Cakmak et al., 1994; Wang et al., 2012), resulting in higher AA accumulation in roots and plant biomass (**Table 1**; **Figure 4**). The change trends of soluble protein content in leaves and roots of sweet potatoes were different under K deficiency. K deficiency reduced protein content in leaves by 2%–33% (**Figure 4C**). In accordance with our results, similar results were observed in cotton leaves and faba bean (*Vicia faba* L.) nodules (Wahab and Abdalla, 1995; Zahoor et al., 2017b). On the contrary, average root protein content increased by 6%–31%, smaller than that in leaves (**Figures 4C, D**), probably because decreased protease activity under K deficiency would lead to a decline in protein degradation rate and a significant increment in protein content (Ali et al., 2019). Reduced AA content and improved protein content in roots indicated that the K deficiency could also change the distribution and conversion of N-containing compounds between AAs and proteins in roots.

NR converts nitrate absorbed by the root into nitrite, which is then combined with NiR and converted into NH_4_^+^ and is the first step of the NO_3_^−^ assimilation pathway (Ali et al., 2019). In this study, even if the transcription levels of *NR* and *NiR* increased, decreased, or remain unchanged, NR and NiR activities in roots decreased under K deficiency (**Figures 7B, D**, **9**), and NO_3_^−^–N will be converted into NH_4_^+^ more quickly in plants, verified by a decrease of NO_3_^−^/NH_4_^+^ ratio under K deficiency in roots (**Supplementary Figure S1**). These results were also demonstrated in *Arabidopsis* roots (Armengaud et al., 2009). As it is known, NH_4_^+^–N is absorbed mainly through the pathway of GS/GOGAT. Previous studies have found that low K declined GS activity and GOGAT activity in crop roots. In this study, K deficiency significantly decreased GS activity and the content of NH_4_^+^ and AA; however, the activity of GOGAT was improved (**Figures 8B, D**, **4B**), even if the transcription levels of *GS* and *GOGAT* increased or remain unchanged (**Figure 9**). Higher GOGAT activity under K deficiency did not increase the synthesis of AAs in roots, indicating that the transformation of AAs into proteins in roots and the AA export from roots to leaves were not inhibited. Moreover, K deficiency increased root protein content, and the decreasing amplitude of AA in leaves was larger than that in roots, which fully verified the above viewpoints. In Ningzishu 1 leaves, the contents of NO_3_^−^–N and NH_4_^+^–N decreased in K-deficiency-treated plants, but the NO_3_^−^/NH_4_^+^ ratio was increased, indicating that more NO_3_^−^ was stored under K deficiency. Therefore, the resistance of NR and NiR activities to K deficiency may be a dominant factor that ameliorates the growth between Xushu 32 and Ningzishu 1 with different low-K sensitivities, in agreement with sweet potato and *Salicornia europaea* under abiotic stress conditions, revealing improved NO_3_^−^ uptake (Nie et al., 2015; Xia et al., 2020).

## Conclusion

**Figure 10:**
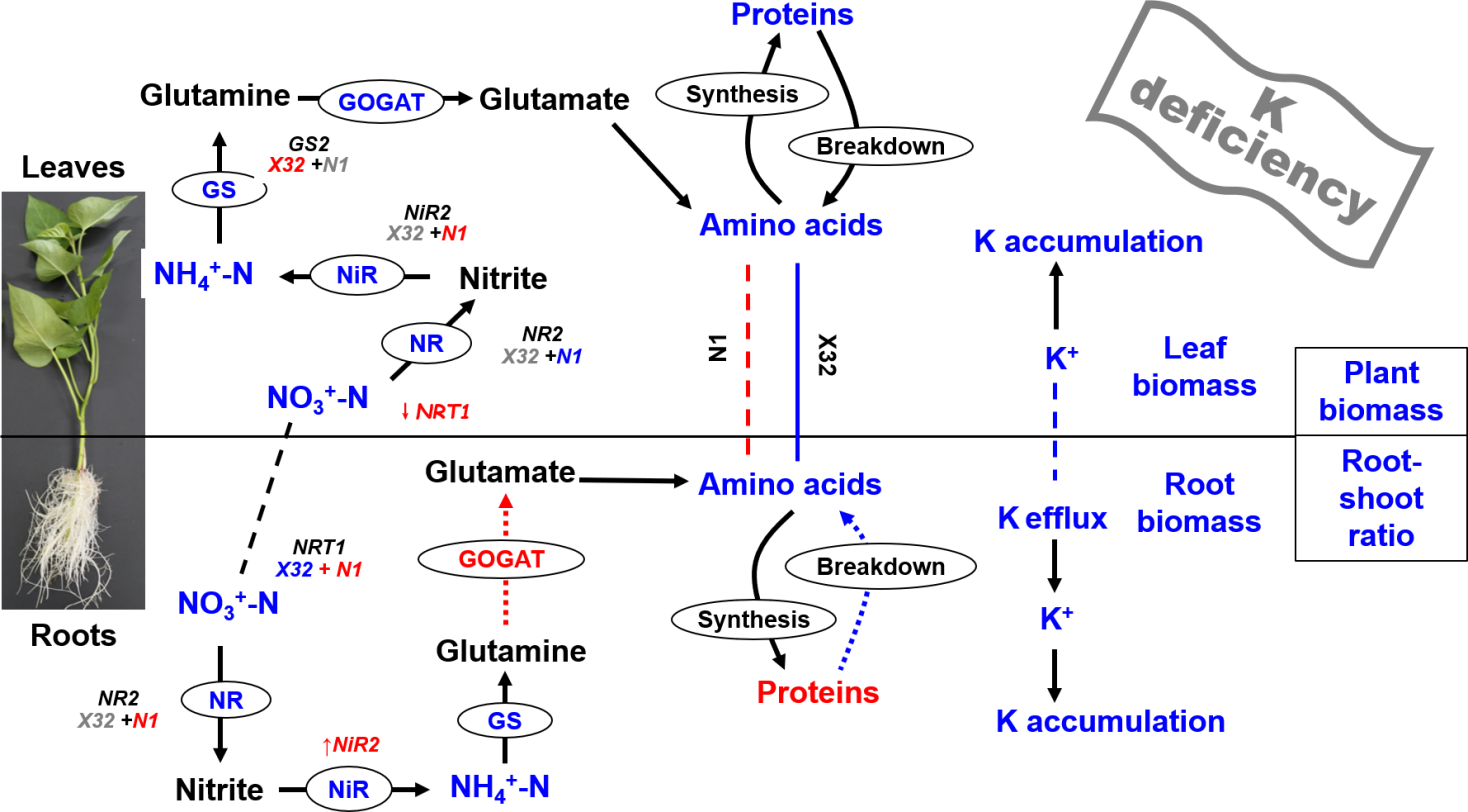
Scheme summarizing the effects of K deficiency on primary metabolism in leaves and roots of sweet potato. Biochemical and transport pathways are indicated with solid and dashed arrows, respectively. Increases in metabolite concentrations and enzyme activities under K deficiency are shown in red, decreases are shown in blue, and items that do not change significantly under K deficiency are marked with gray. Putative direct inhibition under K deficiency is indicated with the red bar. Dashed lines indicate the exchange of metabolites between leaves and roots. X32 and N1 represent Xushu 32 and Ningzishu 1, respectively.

Based on these results, a scheme summarizing the effects of K deficiency on primary metabolism in the leaves and roots of sweet potatoes is proposed (**Figure 10**). In summary, K deficiency significantly decreased the biomass of various organs and whole plants in sweet potatoes, increased K^+^ efflux from roots, and reduced the accumulation of K and N in leaves and roots. In sweet potatoes, leaf K, leaf N, NO_3_^−^–N, and NH_4_^+^–N in leaves and roots declined under K deficiency, and leaf K content had a significant quadratic relationship with soluble protein, NO_3_^−^–N, or NH_4_^+^–N in leaves and roots. Under K deficiency, the transformation of AAs into proteins in roots and the AA export from roots to leaves were not inhibited. Moreover, the resistance of NR and NiR activities to K deficiency may be a dominant factor that ameliorates the growth between Xushu 32 and Ningzishu 1 with different low-K sensitivities.

## Data availability statement

The original contributions presented in this study are included in the article/**Supplementary Material**. Further inquiries can be directed to the corresponding authors.

## Author contributions

JL and ZL conceived and designed the research. HX, YG, and DP performed the research and analyzed the data. JS provided the help of K^+^ flux measurement. JL wrote the paper. ML and ZT provided the plant materials. All authors contributed to the article and approved the submitted version.

## Funding

This work was financially supported by the Natural Science Foundation of the Jiangsu Higher Education Institutions of China (19KJB210012), the earmarked fund (CARS-10-Sweetpotato), the Priority Academic Program Development of Jiangsu Higher Education Institutions (PAPD), and Research and Practice Innovation Project for Postgraduate of Jiangsu Normal University (2022XKT0909).

## Conflict of interest

The authors declare that the research was conducted in the absence of any commercial or financial relationships that could be construed as a potential conflict of interest.

## Publisher’s note

All claims expressed in this article are solely those of the authors and do not necessarily represent those of their affiliated organizations, or those of the publisher, the editors and the reviewers. Any product that may be evaluated in this article, or claim that may be made by its manufacturer, is not guaranteed or endorsed by the publisher.
