# Supplementary material for: Potassium deficiency causes more nitrate nitrogen to be stored in leaves for low-K sensitive sweet potato genotypes
Source: Front Plant Sci. 2022 Nov 23;13:1069181. doi: 10.3389/fpls.2022.1069181 (PMC9764221; doi:10.3389/fpls.2022.1069181)
Supplement: Supplementary file 1 [file DataSheet_1.docx]

Supplementary Table and Figure

**Supplementary Table 1** | Specific primers for gene amplification

| **Enzyme/Protein** | **Target gene** | **Forward primer (5′→3′)** | **Reverse primer (5′→3′)** |
| --- | --- | --- | --- |
| Nitrate  transporter 1.1 | *NRT1.1* | GGTTGAGGCTTGTGAGAGGTT | GTTATTGGCGGCGTTAGCATTC |
| Nitrate reductase | *NR2* | GGAATAACGCCGATCTACCAAG | GAATATCCTCCTCCGTCCGATT |
| Nitrite reductase | *NiR* | TTGGGAGGGAGGATTGGGAG | CATCTTCTTCCCTGTCCCGT |
| Glutamate synthase  [NADH] | *GOGAT* | CAAGGACTTACGAGGTGCTAAC | CGGCTGTCTGTCTCCATGT |
| Glutamine synthetase | *GS2* | GCCTATCCCAACCAACAAGAG | GCTCCAATGCCACAGTAGTATG |
| Glyceraldehyde-3-phosphate dehydrogenase | *GAPDH* | ATACTGTGCACGGACAATGG | TCAGCCCATGGAATCTCTTC |





**Supplementary Figure 1** Effects of K deficiency on the NO_3_^-^/NH_4_^+^ ratio in leaves and roots of the sweet potato
